# Xinyang Tablet attenuates chronic hypoxia-induced right ventricular remodeling via inhibiting cardiomyocytes apoptosis

**DOI:** 10.1186/s13020-022-00689-2

**Published:** 2022-12-05

**Authors:** An-Ran Gao, Shuo Li, Xiao-Cui Tan, Ting Huang, Hua-Jin Dong, Rui Xue, Jing-Cao Li, Yang Zhang, You-Zhi Zhang, Xiao Wang

**Affiliations:** 1 Laboratory Animal Center, Guangzhou University of Chinese Medicine, Guangzhou, 510405 China; 2 State Key Laboratory of Toxicology and Medical Countermeasures, Beijing Key Laboratory of Neuropsychopharmacology, Beijing Institute of Pharmacology and Toxicology, Beijing, People’s Republic of China

**Keywords:** Xingyang Tablet, Hypoxia, Right ventricular remodeling, Cardiomyocytes, Apoptosis

## Abstract

**Background:**

Hypoxia-induced pulmonary hypertension (HPH) is one of the fatal pathologies developed under hypobaric hypoxia and eventually leads to right ventricular (RV) remodeling and RV failure. Clinically, the mortality rate of RV failure caused by HPH is high and lacks effective drugs. Xinyang Tablet (XYT), a traditional Chinese medicine exhibits significant efficacy in the treatment of congestive heart failure and cardiac dysfunction. However, the effects of XYT on chronic hypoxia-induced RV failure are not clear.

**Methods:**

The content of XYT was analyzed by high-performance liquid chromatography-tandem mass spectrometry (HPLC–MS). Sprague–Dawley (SD) rats were housed in a hypobaric chamber (equal to the parameter in altitude 5500 m) for 21 days to obtain the RV remodeling model. Electrocardiogram (ECG) and hemodynamic parameters were measured by iWorx Acquisition & Analysis System. Pathological morphological changes in the RV and pulmonary vessels were observed by H&E staining and Masson’s trichrome staining. Myocardial apoptosis was tested by TUNEL assay. Protein expression levels of TNF-α, IL-6, Bax, Bcl-2, and caspase-3 in the RV and H9c2 cells were detected by western blot. Meanwhile, H9c2 cells were induced by CoCl_2_ to establish a hypoxia injury model to verify the protective effect and mechanisms of XYT. A CCK-8 assay was performed to determine the viability of H9c2 cells. CoCl_2_-induced apoptosis was detected by Annexin-FITC/PI flow cytometry and Hoechst 33,258 staining.

**Results:**

XYT remarkably improved RV hemodynamic disorder and ECG parameters. XYT attenuated hypoxia-induced pathological injury in RV and pulmonary vessels. We also observed that XYT treatment decreased the expression levels of TNF-α, IL-6, Bax/Bcl-2 ratio, and the numbers of myocardial apoptosis in RV. In H9c2 myocardial hypoxia model, XYT protected H9c2 cells against Cobalt chloride (CoCl_2_)-induced apoptosis. We also found that XYT could antagonize CoCl_2_-induced apoptosis through upregulating Bcl-2, inhibiting Bax and caspase-3 expression.

**Conclusions:**

We concluded that XYT improved hypoxia-induced RV remodeling and protected against cardiac injury by inhibiting apoptosis pathway in vivo and *vitro* models, which may be a promising therapeutic strategy for clinical management of hypoxia-induced cardiac injury.

## Introduction

Many people live permanently at a high altitude and approximately 40 million individuals are exposed to high altitudes for hours or days [1, 2]. Hypoxia-induced pulmonary hypertension (HPH) is one of the fatal pathologies developed under hypobaric hypoxia [3]. In the face of chronic hypoxia, the pulmonary vessels adapt by vasoconstriction and remodeling, leading to the development of HPH and an increasingly larger load on the right ventricle, eventually causing right ventricular (RV) remodeling and RV failure [4–6]. RV remodeling is featured by initially adaptive hypertrophy but may be ultimately decompensated, as evidenced by dilatation, fibrosis, and failure [6, 7]. Though the initial insult of HPH is the pulmonary vasculature, RV failure remains a leading cause of mortality in patients with HPH [8, 9]. The current clinical medications for HPH act primarily on the pulmonary vasculature, with only secondary effects on the RV [10]. Therefore, how to control or even reverse RV dysfunction has gained more and more attention recently.

Although increased RV pressure overload is the first trigger for RV adaption, apoptosis and inflammation may contribute to the transition toward RV dilatation and failure [11, 12]. Clinical trials are seeking for more effective strategies for prevention of RV failure in HPH. Previous studies demonstrated that apoptosis plays an important role in the cardiac dysfunction and structural changes in the process of RV remodeling and development of subsequent heart failure [13, 14]. And also, cardiomyocyte apoptosis has been recognized as a significant cause of cardiac hypertrophy and can be served as a predictive factor for the severity of adverse remodeling and the occurrence of heart failure [15, 16]. Therefore, anti-apoptosis has been believed as an attractive approach for RV failure intervention [17]. Besides, several studies demonstrated that the pro-inflammatory cytokines initiated by hypoxia, such as tumor necrosis factor-alpha (TNF-α), interleukin-6 (IL-6) and interleukin-1β (IL-1β), which might lead to right ventricular hypertrophy (RVH), cardiac fibrosis and failure and might also be biomarkers in RV failure.

Traditional Chinese medicine (TCM), as a complementary and alternative approach, has been widely used in the treatment of cardiovascular disease in China. In terms of TCM, heart failure primarily ascribes to the pathological products of Qi and Yang deficiency, blood stasis, and fluid stagnation. The therapy of ‘Wenyang, Yiqi, Huoxue’ has been recognized as the basic principle of heart failure. Previous studies reported that this therapy improved left ventricular ejection fraction with heart failure, inhibited cardiac hypertrophy, and ameliorated the symptoms of chronic pulmonary heart disease induced by chronic obstructive pulmonary disease [18–20]. XYT is a commercial product approved by the Guangdong Pharmaceutical and Food Administration (No. Z20071257). It is traditionally used to treat heart failure associated with Qi-Yang deficiency through benefiting Qi and warming Yang, activating blood circulation, and inducing diuresis [21]. Clinically, XYT has gained widespread applications in the treatment of congestive heart failure [22]. Previous studies found that XYT improved left ventricular ejection fraction with heart failure [23–25]. However, it remains unknown whether XYT plays a role in the pathogenesis and progression of chronic hypoxia-induced RV remodeling. In the present study, we investigate the effects of XYT on the structure and function of RV in a rat model of chronic hypoxia and the possible underlying mechanisms in vivo and *vitro*.

## Materials and methods

### Drug and reagents

XYT includes seven traditional Chinese herbs: *Astragalus membranaceus* (Huang Qi), *Epimedium sagittatum* (Yin Yang Huo), *Radix Ginseng Rubra*, (Hong Shen), *Leonurus japonicus* (Yi Mu Cao), *Ilex pubescens *(Mao Dong Qing), *Descurainiae Semen* (Ting Li Zi), and *Plantago asiatica* (Che Qian Zi), all of which are recorded in the Chinese Pharmacopoeia. XYT (No. 20200901) was purchased from The First Affiliated Hospital of Guangzhou University of Chinese Medicine and approved by the Guangdong Pharmaceutical and Food Administration (No. Z20071257). The quality control of XYT has been established according to previous studies [21]. Captopril (Cap) was obtained from Sigma (USA). The following antibodies were purchased from CST (USA): anti-IL-6, anti-Bcl-2, anti-Bax, anti-TNF-α, and anti-caspase-3. All the other chemicals and reagents were of standard commercially available biochemical quality.

### HPLC–MS analysis

We dissolved XYT powder (0.25 g) in 30 ml methanol–water solution (60%, v/v), mixed it evenly, ultrasonicated the mixture for 30 min, and centrifuged it at 5000 r/min for 10 min. Then the supernatant was diluted 10 times and passed through a 0.22 μm microporous filter membrane. We injected aliquots of XYT solution into a UPLC I-Class HPLC system (Waters Corporation, USA) for analysis. All components were separated on Acquity UPLC^®^ HSS T3 column (100 mm × 2.1 mm, 1.8 μm), column temperature was 45 ℃ and flow rate was 0.3 ml/min. The mobile phase was composed of (A) a formic acid aqueous solution (0.1%, v/v) and (B) acetonitrile using a gradient elution of 90%–80% A at 0–7 min, 80%–65% A at 7–14 min, 65%–50% A at 14–21 min, 50%–35% A at 21–28 min, 35%–10% A at 28–35 min, 10% A at 35–37 min, 10%–90% A at 37–37.1 min, and 90% A at 37.1–40 min. Mass spectrometry was performed on a XEVO TQ-S Micro quadrupole time-of-flight mass spectrometry (Q-TOF/MS)(Waters Corporation, USA). Ion source temperature was 100 ℃, dissolvent gas temperature was 550 ℃ and flow rate was 600 l/h, air flow rate in conical hole was 50 l/h, capillary voltage was 2 kV, taper hole voltage was 40 eV, collision energy was 20–40 eV. The scan range was 50–1200 m/z.

### Animals

Male Sprague–Dawley (SD) rats (weight 250–300 g) were purchased from the SPF Biotechnology (Beijing, China). All animals were housed under controlled temperature and humidity with a 12 h light/12 h dark cycle. Rats were allowed free access to food and water. For the chronic hypoxia animal model, rats were maintained for 21 days at an equivalent altitude of 5500 m in a hypobaric chamber (Guizhou Feng Lei Oxygen Chamber). Rats were treated with consecutive XYT (270 mg/kg, i.g.) or Cap (30 mg/kg, i.g.) once a day for 21 days. According to the 70 kg adult clinical dose of XYT (3 g per day), equivalent dose of 270 mg/kg was used in this study. It was also an effective dose for cardioprotective effects, based on the previous study [21].

### Electrocardiogram (ECG) and hemodynamic measurements

Rats were anesthetized with sodium pentobarbital (30 mg/kg, i.p.) and electrodes were inserted under the skin for the limb lead at position II. ECG was then recorded (P wave amplitude and QT interval). Next, a catheter filled with heparin saline (500 U/mL) was inserted into the right ventricle through the right jugular vein, and then RV systolic pressure (RVSP), RV end-diastolic pressure (RVEDP) as well as the maximal rate of increase and decrease in RV pressure (+ dP/dtmax and -dP/dtmin, respectively) were measured by an IX/416 data acquisition system (Kaha Sciences Ltd, New Zealand).

### RVH assessment and samples collection

After recording ECG and hemodynamic parameters, the rats were sacrificed and the heart and lung tissues were harvested. Then the right ventricle and the left ventricle plus interventricular spetum (LV + S) were weighted. The RVH index (RVHI) was measured as: RVHI (%) = [RV / (LV + S)] × 100% [26]. The ratio of RV weight to body weight (RVW / BW) was also calculated. The RV tissues were horizontally cut into two parts. The upper one half of the RV tissues and the right lower pulmonary lobes were fixed in 4% paraformaldehyde for histological analysis. And the remaining half of RV tissues was stored at − 80 °C for western blot analysis.

### Histological analysis

The morphological changes in the right ventricle and pulmonary vessels were stained using hematoxylin and eosin (HE) staining. The percentage of medial wall thickness (WT%) and medial wall area (WA%) were calculated to determine the extent of pulmonary vascular structure remodeling (medial wall hypertrophy): WT% = [(external vessel diameter—internal vessel diameter)/(external vessel diameter)] × 100%; WA% = [(total vessel area—luminal vessel area)/(total vessel area)] × 100%. Masson’s trichrome staining was performed to assess the degree of fibrosis (collagen fibers stained blue) in the RV and lung tissues [27]. TUNEL staining was used to detect cardiomyocyte apoptosis. All data were quantified and analyzed using Image-Pro Plus version 6.0 software.

### Protein preparation and western blot analysis

Cells or heart tissue from each group were lysed using RIPA buffer. The lysate was centrifuged at 12,000 rpm and 4 °C for 20 min, and the supernatant was harvested. The protein concentration was detected by BCA protein assay kit. Western blot analysis was performed according to standard protocols with slight modifications. The equal amounts of proteins (40 µg) were separated using SDS-PAGE and transferred onto PVDF membranes. Next, the membranes were blocked with 5% non-fat milk. The membranes were incubated with antibodies overnight at 4 °C and then incubated with secondary antibodies. Analysis of the protein bands was performed using AlphaView SA software.

### H9c2 Cell culture and CoCl_2_ (Cobalt chloride)-induced hypoxia model

The H9c2 cell line was purchased from Procell Life Science&Technology Co.,Ltd. (Wuhan, China). The H9c2 cells were cultured in high-glucose Dulbecco's modified Eagle's medium (Gibco, USA) containing 10% fetal bovine serum (Gibco, USA) and 1% antibiotics penicillin/streptomycin (Gibco, USA) in an incubator at 37 ºC with 5% CO_2_. The medium was changed every 2–3 days.

The H9c2 cells were exposed to 600 μmol/L of Cobalt chloride (CoCl_2_) in the culture medium for 24 h to induce cell hypoxia as previous studies described [28]. Then, different concentrations (0, 5, 10, 30, and 60 µg/ml) of XYT were used to treat H9c2 cells for 24 h. The control cells were incubated without XYT or CoCl_2_. A CCK-8 assay was performed to determine the viability of H9c2 cells. Cells were seeded into 96-well plates at 5 × 10^3^ cells/well with complete medium. After exposure to various treatments, 10 µl CCK8 reagent (Dojindo Molecular Technologies, Inc.) and 100 µl DMEM was then added. The plates were incubated at 37 ºC for 2 h. The absorbance was measured using a microplate reader (Berthold Group, Ltd., Wildbad, Germany) at 450 nm.

### Flow cytometry and Hoechst staining

Cell apoptosis analysis was evaluated using Annexin V-FITC/PI apoptosis detection kit (Beyotime, Shanghai, China). After various treatments, cells were collected into tubes. Then the cells were re-suspended and incubated with 5 μl Annexin V and 5 μl propidium iodide (PI) in the dark for 1 h at room temperature. Flow cytometry analysis was detected using a flow cytometer (BD, FACSCalibur Flow Cytometer).

The extent of apoptosis was also determined by the morphologic changes in cell nuclei by using Hoechst staining and examined under a fluorescence microscope (Beyotime, Shanghai, China). Apoptosis index = apoptotic nuclei amount/total nuclei amount × 100%.

### Statistical analysis

All the values were expressed as the mean ± standard error of the mean (SEM) of multiple independent replicates. Statistical analyses were conducted using one-way analysis of variance (ANOVA). All analyses were performed with GraphPad Prism Version 8.2.1.

## Results

### HPLC–MS analysis of Xinyang Tablet

**Fig. 1:**
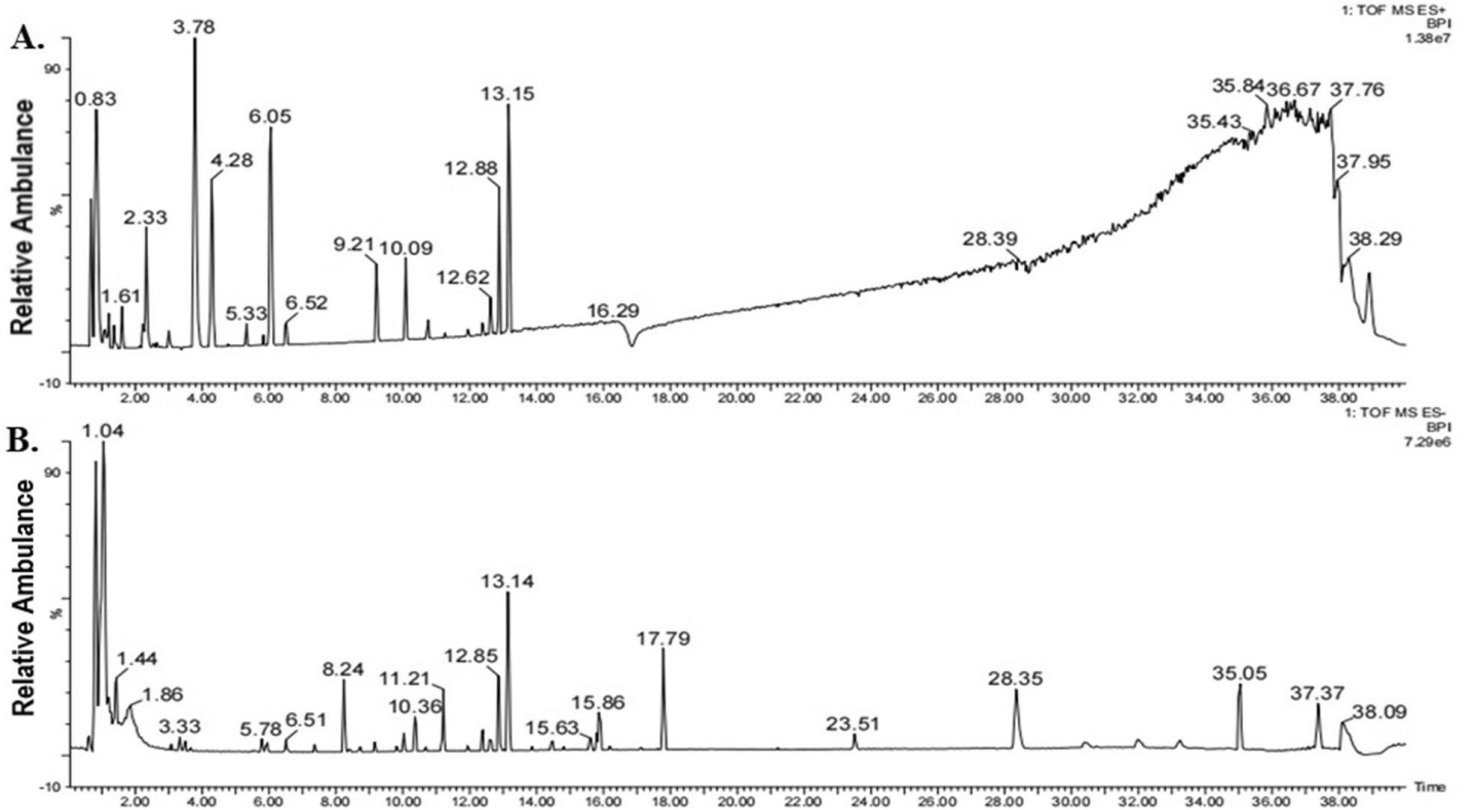
The chromatographic fingerprint of XYT. **A** Ion chromatograms chromatogram in positive electrospray ionization mode. **B** Ion chromatograms chromatogram in negative ion mode. Structures of these compounds are listed in Table 1

Q-TOF/MS was used to match the active components in the database to those in XYT. The results of chemical composition identification are shown in Table 1. And the Ion flow diagrams of XYT in positive ion mode and negative ion mode are shown in Fig. 1 and Table 1.

**Table 1:** Formulary identification of the chemical constituents of XYT as determined by HPLC–MS analysis

Number	t_R_/min	Name	Formula	Scanning Mode	MS(m/z)	δ/mDa	Adduct Ion
1	0.85	Stachydrine	C_7_H_13_NO_2_	ESI+	144.1008	− 1.1	+H
2	1.43	Geniposidic acid	C_16_H_22_O_10_	ESI −	373.1140	0.0	− H
3	2.14	Quercetin	C_15_H_10_O_7_	ESI+	303.0482	− 1.8	+H
4	3.78	Baohuoside II	C_26_H_28_O_10_	ESI+	251.0906	0.0	+2H
5	5.32	Leonurine	C_14_H_21_N_3_O_5_	ESI+	312.1545	− 0.9	+H
6	6.51	Calycosin-7-O-β-d-glucoside	C_22_H_22_O_10_	ESI −	491.1196	0.1	+HCOO
7	8.23	Verproside	C_29_H_36_O_15_	ESI −	623.1974	− 0.7	− H
8	8.71	Syringaresinol	C_22_H_26_O_8_	ESI −	417.1530	− 2.5	− H
9	9.13	Plantamajoside	C_29_H_36_O_16_	ESI −	639.1931	0.0	− H
10	11.19	Ginsenoside Rd	C_48_H_82_O_18_	ESI −	991.5493	0.9	+HCOO
11	11.94	Calycosin	C_16_H_12_O_5_	ESI+	285.0742	− 1.5	+H
12	12.62	Sagittatoside B	C_32_H_38_O_14_	ESI −	645.2188	− 0.1	− H
13	12.89	Epmedin C	C_39_H_50_O_19_	ESI+	823.3022	0.3	+H
14	13.18	Icariin	C_33_H_40_O_15_	ESI+	677.2443	0.3	+H
15	14.46	Ginsenoside Rg1	C_42_H_72_O_14_	ESI −	845.4918	1.4	+HCOO
16	15.60	Ginsenoside Rb1	C_54_H_92_O_23_	ESI −	1107.5974	1.8	− H
17	15.79	20(R)-Ginsenoside Rh1	C_36_H_62_O_9_	ESI −	683.4382	0.7	+HCOO
18	15.89	Ginsenoside Ro	C_48_H_76_O_19_	ESI −	955.4917	0.9	− H
19	17.06	Isoastragaloside IV	C_41_H_68_O_14_	ESI −	829.4590	− 0.1	+HCOO
20	17.80	Ilexsaponin A1	C_36_H_56_O_11_	ESI −	663.3757	0.7	− H
21	23.53	Ilexgenin A	C_30_H_46_O_6_	ESI −	501.3224	0.2	− H

### XYT attenuated RV hyperfunction and hemodynamic disorder in rats

**Fig. 2:**
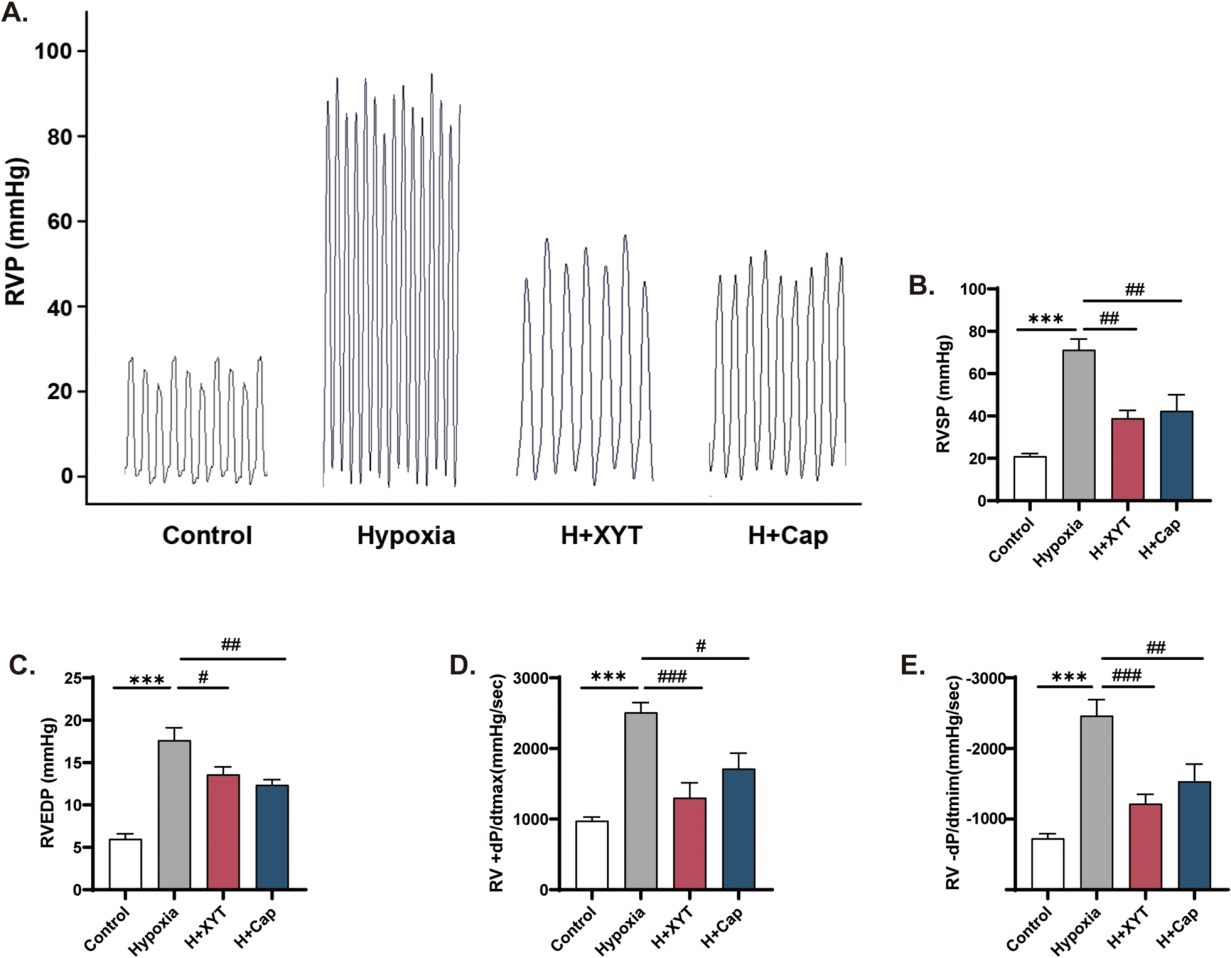
The effects of XYT on hemodynamic disorder in rats. **A** Representative images of right ventricular pressure (RVP). **B**–**E** Quantitative analysis of right ventricular systolic pressure (RVSP), right ventricular end-diastolic pressure (RVEDP), the maximal rate of increase (+ dP/dtmax) and decrease (-dP/dtmin) in RVP. n = 6; *** *p* < 0.001 vs. control group; ^#^*p* < 0.05, ^##^*p* < 0.01, ^###^*p* < 0.001 vs. hypoxia group. Statistical analyses were conducted using one-way analysis of variance (ANOVA)

As shown in Fig. 2B and C, RVSP and RVEDP were increased in hypoxia group when compared with control group (*p* < 0.001). Furthermore, increased RV + dP/dtmax and decreased RV -dP/dtmin were observed under hypoxia exposure, which may be associated with RV hyperfunction and hypercontractile remodeling (*p* < 0.001, Fig. 2D and E). After treatment with XYT or Cap, RVSP, RVEDP, and RV + dP/dtmax were significantly decreased, whereas RV -dP/dtmin was obviously increased (*p* < 0.05, *p* < 0.01, *p* < 0.001). Hemodynamic examinations indicated that XYT could suppress RV hyperfunction and hemodynamic disorder induced by hypoxia exposure.

### XYT ameliorated ECG parameters in rats

**Fig. 3:**
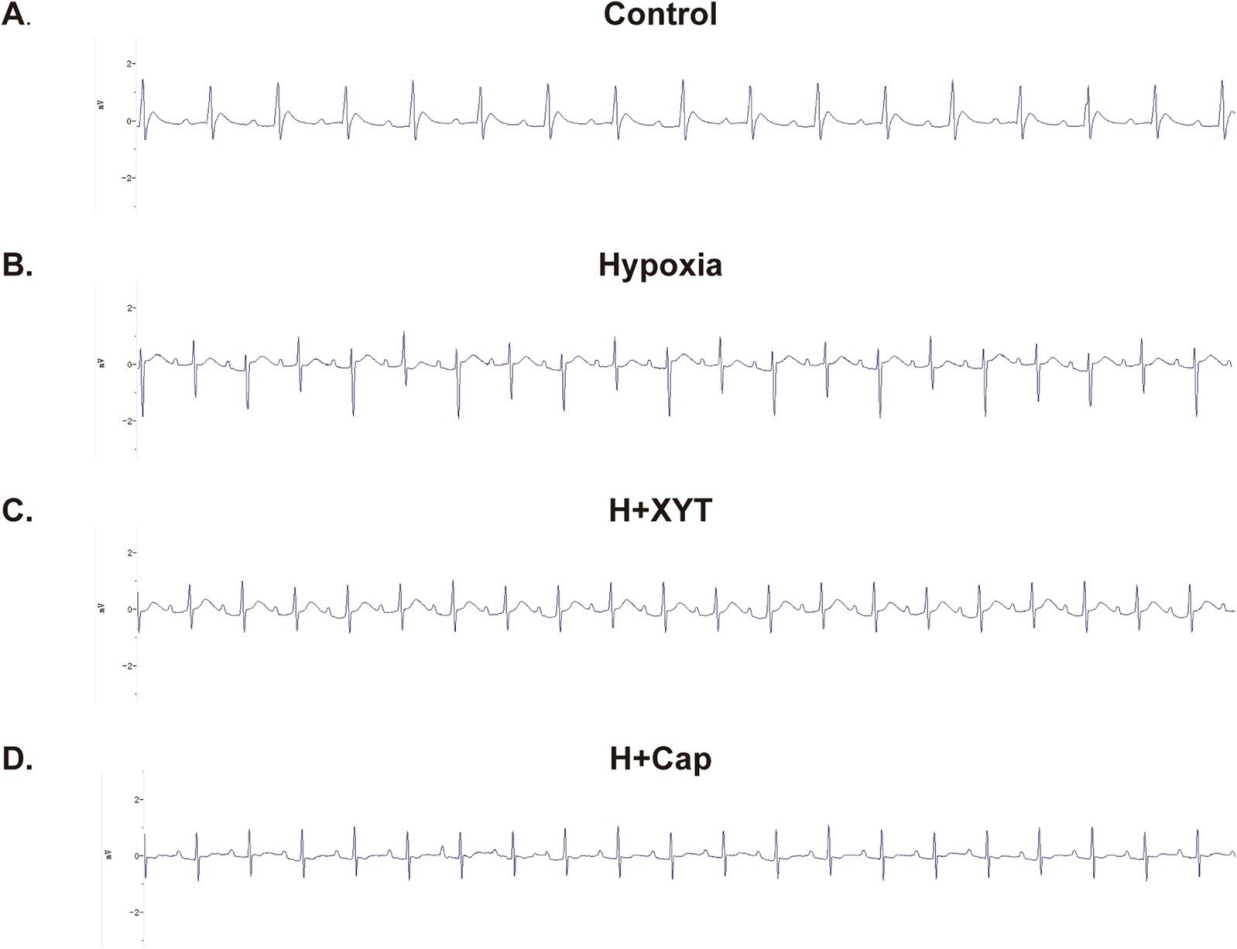
Representative images of ECG in rats. Electrocardiogram recordings in anesthetized mice using lead II. The electrocardiograms **A**–**D** were control, hypoxia, hypoxia treated with XYT (270 mg/kg) and hypoxia treated with captopril (30 mg/kg) groups, respectively. The heart rate, P wave amplitude and QT interval were recorded

ECG is currently one of the main tests used to diagnose abnormal cardiac function. As shown in Fig. 3 and Table 2, compared with the control group, the hypoxia group had higher P wave amplitude, prolonged QT, and rate-corrected QT (QTc) intervals in lead II, which may be related to pulmonary hypertension accompanied by RVH (*p* < 0.05, *p* < 0.001). However, XYT-treated rats exhibited lower P wave amplitude, and shorten QTc interval (*p* < 0.01, *p* < 0.001, Table 2) but not QT interval compared to the hypoxia group. Treatment with Cap was able to decrease QTc interval (*p* < 0.05) but not P wave amplitude and QT interval. There was no significant difference in heart rate among different groups. These data demonstrated that XYT could improve ECG parameters in RVH rats.

**Table 2:** The effects of XYT on ECG parameters

ECG parameters	Control	Hypoxia	H+XYT	H+Cap
Heart rate (beat/min)	309.100 ± 14.410	388.700 ± 37.380	309.100 ± 12.610	343.400 ± 8.084
P Amp. (mv)	0.124 ± 0.015	0.227 ± 0.025*	0.130 ± 0.029^##^	0.244 ± 0.018
QTc interval (ms)	135.200 ± 5.389	241.600 ± 4.596***	202.100 ± 4.766^###^	217.500 ± 5.015^#^
QT interval (ms)	52.100 ± 2.709	98.330 ± 0.717***	91.070 ± 1.268	91.970 ± 2.772

P Amp. indicated amplitude of the P wave. QTc interval indicated rate-corrected QT Interval. n = 6, *p < 0.05, *** *p* < 0.001 vs. control group; ^#^*p* < 0.05, ^##^*p* < 0.01, ^###^*p* < 0.001 vs. hypoxia group. Statistical analyses were conducted using one-way analysis of variance (ANOVA)

### XYT relieved RV and pulmonary vascular remodeling in rats

**Fig. 4:**
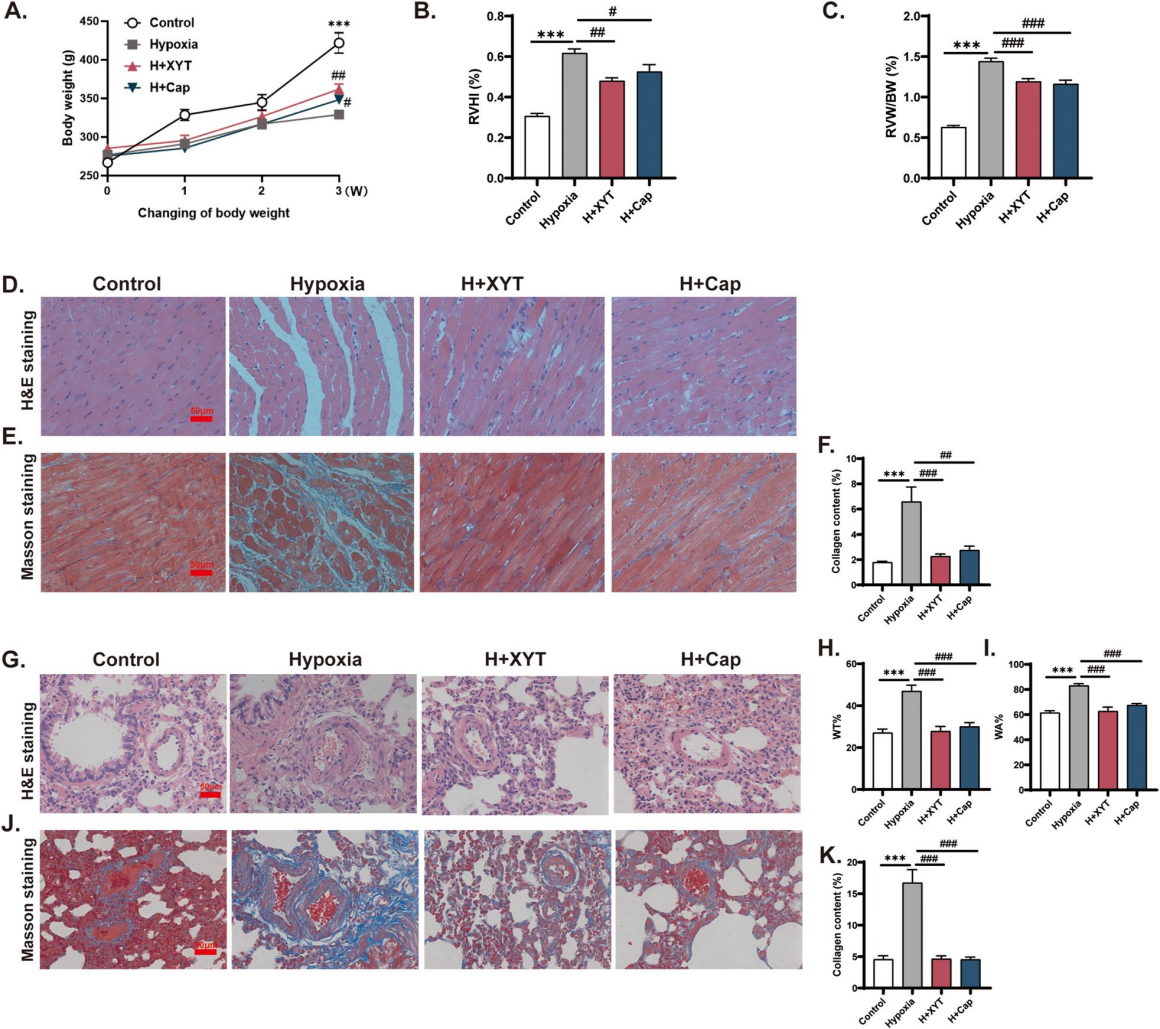
The effects of XYT on hypoxia-induced RV and pulmonary vascular remodeling in rats. **A** Time-dependent body weight in different groups (n = 7). **B** RVHI: the ratio of right ventricular weight to left ventricular weight plus septum weight (RV/(LV + S) × 100%). **C** RVW/BW: the ratio of RV weight to body weight. **D** Representative micrographs stained with H&E staining in the RV. **E** Masson's trichrome staining (collagen fibers were stained blue) in the RV. Original magnification × 400, scale bar = 50 μm. **F** Quantitation of collagen content in the RV. **G** H&E staining in the pulmonary arteries. **H** WT%: the percentage of medial wall thickness. **I** WA%: the percentage of medial wall area. **J** Masson's trichrome staining in the pulmonary arteries. Original magnification× 400, scale bar = 50 μm. **K** Quantitation of lung perivascular collagen content. n = 6; ***p < 0.001 vs. control group; ^#^*p* < 0.05, ^##^*p* < 0.01, ^###^*p* < 0.001 vs. hypoxia group. Statistical analyses were conducted using one-way analysis of variance (ANOVA)

As shown in Fig. 4A, the BW of hypoxia-treated rats was significantly lower than those of controls after 3 weeks (*p* < 0.001). The levels of RVHI and RVW/BW were significantly increased in hypoxia group, indicative of cardiac hypertrophy (*p* < 0.001, Fig. 4B and C). However, daily treatment with XYT or Cap significantly attenuated hypoxia-induced growth retardation and cardiac hypertrophy. RV remodeling is associated with both variations of cardiac morphology and increased interstitial fibrosis (increased myocardial collagen content). Thus, we conducted H&E staining and Masson’s trichrome staining to determine the cardioprotective effects of XYT against RV remodeling. We found that myocardial fibers of RV tissues were markedly thickened, sparsely distributed, occasionally ruptured and dissolved in hypoxia group as compared with control group (Fig. 4D). Masson’s trichrome staining showed that there was excessive collagen obviously deposited in RV tissues of hypoxia rats, as indicated by collagen content (*p* < 0.001, Fig. 4E and F). In contrast, XYT or Cap significantly reversed these adverse morphological changes (*p* < 0.05, *p* < 0.01, *p* < 0.001). These results demonstrated that XYT could prevent further aggravation of RV remodeling.

Pulmonary histology was subsequently performed to investigate the structural alternations that may contribute to an increase in RV afterload. As determined by H&E staining, we found the levels of WT% and WA% in hypoxia group were significantly greater than those in control groups (*p* < 0.001, Fig. 4G, H and I). Masson’s trichrome staining showed the lung perivascular collagen content was elevated in hypoxia-induced rats (*p* < 0.001, Fig. 4J and K). After treatment with XYT or Cap, the levels of WT%, WA%, and collagen content in the small pulmonary arteries were significantly decreased (*p* < 0.001). These results indicated that XYT could ameliorate hypoxia-induced pulmonary vascular remodeling and fibrosis in rats.

### XYT suppressed myocardial apoptosis and inflammation in RV

**Fig. 5:**
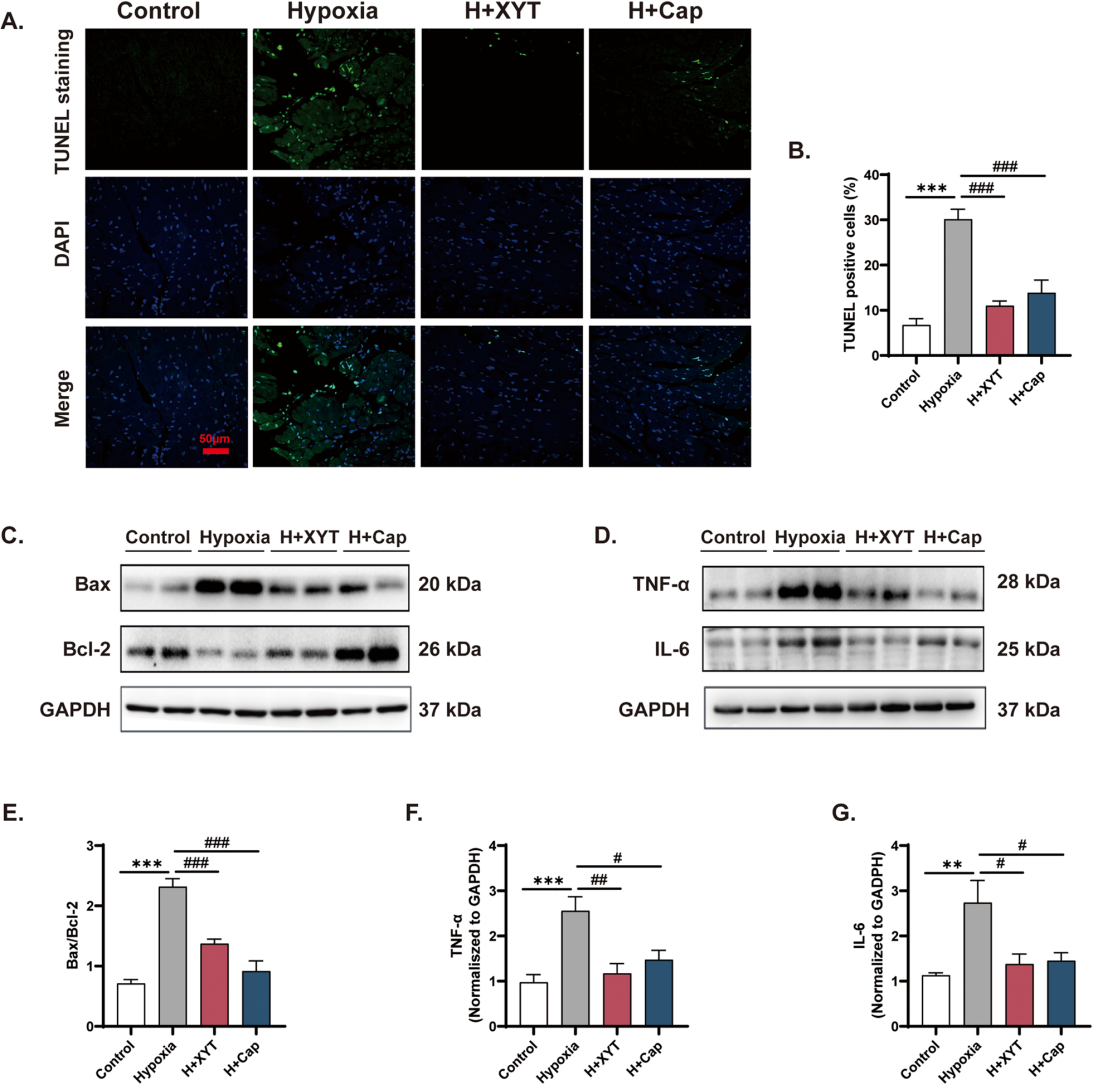
The effects of XYT on myocardial apoptosis and inflammation in rats. **A** Representative images of TUNEL (green spots) in the right ventricle, original magnification × 400, scale bar = 50 μm. **B** The percentage of TUNEL-positive cells (n = 4). **C** and **D** The expression of Bax, Bcl-2, TNF-α, and IL-6 proteins in the right ventricle. **E**–**G** Quantitative analysis of the expression levels of Bax/Bcl-2, TNF-α, and IL-6. n = 6, **p < 0.01, ***p < 0.001 vs. control group; ^#^*p* < 0.05, ^##^*p* < 0.01, ^###^*p* < 0.001 vs. hypoxia group. Statistical analyses were conducted using one-way analysis of variance (ANOVA)

To evaluate the effects of XYT on cardiomyocyte apoptosis induced by hypoxia, TUNEL staining and Western blot were performed. As shown in Fig. 5A and B, TUNEL-positive cells of hypoxia rats were significantly increased compared with controls (*p* < 0.001). Western blot analysis indicated that the ratio of Bax/Bcl-2 in hypoxia group was higher than that in control group (*p* < 0.001, Fig. 5C and E). Impressively, treatment with XYT or Cap effectively reversed hypoxia-induced increases in TUNEL-positive cells and Bax/Bcl-2 ratio in the right ventricle (*p* < 0.001). In addition, we found that the expression levels of TNF-α and IL-6 proteins in the right ventricle were increased under hypoxic conditions, which were attenuated in XYT- and Cap-treated rats (*p* < 0.05, *p* < 0.01, *p* < 0.001, Fig. 5D, F and G). Overall, these findings suggested that XYT preserved RV remodeling by blunting myocardial apoptosis and inflammation.

### XYT protects H9c2 cells against apoptosis induced by CoCl_2_ treatment

**Fig. 6:**
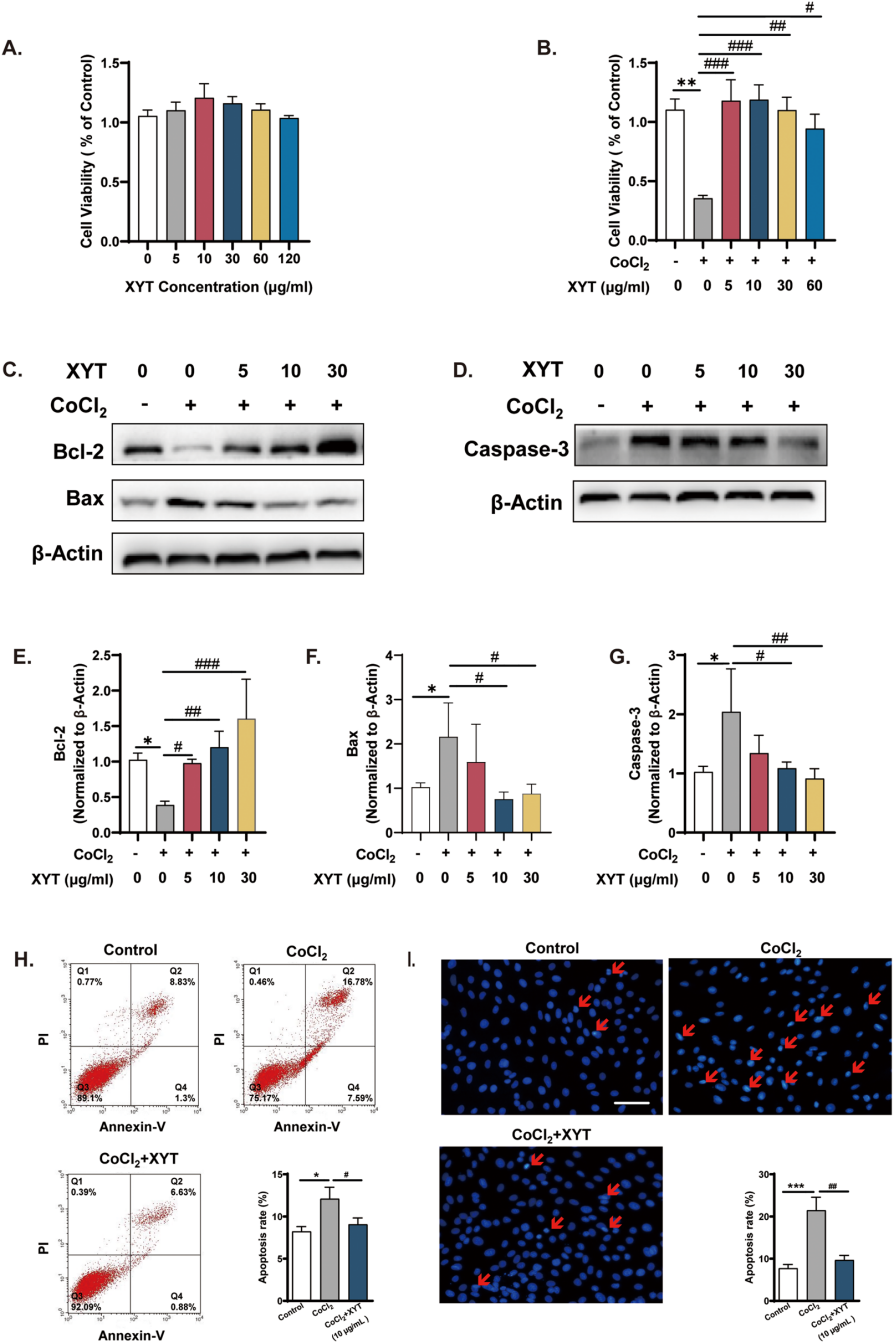
XYT protected H9c2 cells against CoCl_2_-induced cytotoxicity. **A** and **B** The effects of different concentrations of XYT on cell viability were evaluated in H9c2 cells and CoCl2 treatment of H9c2 cells. **C** and **D** The expression of Bax, Bcl-2, and caspase-3 proteins in cells. **E**–**G** Quantitative analysis of the expression levels of Bcl-2, Bax, and caspase-3. **H** and **I** Cell apoptosis was evaluated using flow cytometry and Hoechst 33,258 staining assay, original magnification × 400, Scale bars = 100 µm. n = 3–6, *p < 0.05, **p < 0.01 vs. control group; ^#^*p* < 0.05, ^##^*p* < 0.01, ^###^*p* < 0.001 vs. hypoxia group. Statistical analyses were conducted using one-way analysis of variance (ANOVA)

Based on the above results, the myocardial protective effects of XYT were next studied in vitro. To investigate the effects of XYT on hypoxia-induced cell injury, CoCl_2_ was used to mimic the hypoxia condition. As shown in Fig. 6A, XYT alone did not interfere with the cell viability of H9c2 cells in different concentrations. In addition, XYT treatment improved the viability in CoCl_2_-treated cells in a concentration dependent manner (*p* < 0.05, *p* < 0.01, *p* < 0.001, Fig. 6B). To examine the effects of XYT on apoptosis, the expression levels of Bax, Bcl-2, and caspase-3 were detected. CoCl_2_-induced hypoxic injury downregulated Bcl-2 and upregulated Bax expression, compared to control (*p* < 0.05, Fig. 6C–F). With XYT pretreatment, the expression of Bcl-2 was gradually decreased and the expression of Bax was increased in a dose-dependent manner (*p* < 0.05, *p* < 0.01, *p* < 0.001). Moreover, the expression level of another pro-apoptotic marker, caspase-3 was markedly higher in CoCl_2_ group than in the control group (*p* < 0.05, Fig. 6G), while XYT significantly decreased the expression of caspase-3 in a dose-dependent manner (*p* < 0.05, *p* < 0.01).

The effects of XYT on CoCl_2_-induced apoptosis in H9c2 cells were investigated by Annexin-FITC/PI flow cytometry and Hoechst 33,258 staining. As shown in Fig. 6H, the myocardial apoptosis rate was increased to 12.10 ± 1.37% (*p* < 0.05) in the CoCl_2_ group as compared to that in the control group (8.25 ± 0.58%). This increase was significantly attenuated by XYT treatment, with the apoptosis rate 9.06 ± 0.76% (*p* < 0.05) compared to the CoCl_2_ group. Similar results were obtained by Hoechst 33,258 staining (Fig. 6I). Cultures treated with XYT showed a decrease in the apoptosis ratio 9.67 ± 1.1% (*p* < 0.05) compared to the CoCl_2_ group (21.47 ± 3.08%). These results suggested that XYT could protect H9c2 cells against CoCl_2_-induced apoptosis and necrosis.

## Discussion

The major findings of this study revealed that XYT could alleviate chronic hypoxia-induced RV and pulmonary vascular remodeling through attenuation of apoptosis and inflammation. We also provide significant insight into protective effects of XYT on modulation of CoCl_2_-induced hypoxia and apoptotic death via inhibiting apoptotic proteins in H9c2 cardiomyocytes.

In the present study, the animal model of HPH was provided to evaluate the effects of XYT on hypoxia-induced RV remodeling. Chronic hypoxia causes pulmonary vasoconstriction and vascular remodeling, which is considered to be a pathological hallmark during the progression of HPH [29, 30]. These changes in pulmonary vessels will increase RV overload and lead to RV remodeling [4, 31]. Clinically, there is a lack of specific drugs for the treatment of chronic hypoxia-induced right heart injury. Cap is used for treatment of congestive heart failure in clinical practice [32], which also found the same character in XYT. Cap, as a competitive angiotensin-converting enzyme inhibitor, can effectively reduce cardiac load in the treatment of cardiac failure. Previous studies demonstrated that Cap could prevent cardiomyocytes from apoptosis and inflammation [33, 34]. Therefore, we selected Cap as a positive control to evaluate the effects of XYT on RV remodeling. We found that chronic hypoxia caused increasing in RVSP, RVEDP, pulmonary vascular collagen content, and thickening of pulmonary arteriole in rats, which all resemble the characteristics of animal models with HPH [12, 35, 36]. These aberrant changes were effectively prevented by treatment with XYT, indicating that it exerted protective effects against RV remodeling by alleviation of RV workload and pulmonary vascular remodeling.

RV remodeling that occurs with HPH includes RVH and RV fibrosis. During the early stages of HPH, RV remodeling, distinguished by hypertrophic and hypercontractile remodeling, represents a compensatory response to increased afterload [37, 38]. However, with increasing cardiac overload, the adaptive response will gradually shift to RV hyperfunction, maladaptive remodeling, and towards RV failure, which is accompanied by myocardial hypertrophy, apoptosis, and fibrosis [15]. Studies have also confirmed that cardiac hyperfunction leads to cardiac remodeling in the failing heart via increased energy consumption [39]. In the present study, the hypoxia rats displayed cardiac hyperfunction (increased RV + dP/dtmax and decreased RV -dP/dtmin). In ECG, increased P wave amplitude and prolonged QTc intervals were observed in hypoxia group. These changes have a diagnostic value in pulmonary hypertension with RVH [40, 41]. Consisted with this, we found that chronic hypoxia increased the levels of RVHI and RVW/BW, indicating that hypoxia was a contributor to RVH. Also, the hypoxia group showed RV fibrosis, characterized by elevation of interstitial collagen content in RV [42]. XYT significantly reduced the RV + dP/dtmax and RV -dP/dtmin, improved the ECG parameters and inhibited the RVHI, RVW/BW, and collagen content in the right ventricle of hypoxia rats. These findings suggested that XYT alleviated hypoxia-induced RV remodeling and hyperfunction.

It is well established that cardiomyocyte apoptosis participates in the pathogenesis of cardiac hypertrophy induced by pressure overload, which further facilitates the transition from adaptive hypertrophy to the maladaptive stage [43, 44]. And hypoxia process may activate the apoptotic pathway, which is regulated by Bcl-2 family proteins. Mitochondrial apoptosis is mediated by the accumulation of pro-apoptotic protein Bax and the reduction of anti-apoptotic protein Bcl-2 [45]. An increase in Bax/Bcl-2 ratio can reflect the activation of the caspase program and the induction of apoptosis [43, 46]. Our results showed that myocardial cell apoptosis was inhibited by XYT treatment through the reduction of Bax/Bcl-2 ratio. In addition, accumulating evidence revealed that inflammation was another trigger factor of RV remodeling [47]. Previous studies reported that inflammatory factor, such as TNF-α and IL-6, cause cardiac hypertrophy and fibrosis through regulate the cardiac inflammatory response [48–51]. We found that XYT could inhibit inflammation by decreasing the TNF-α and IL-6 expression levels in the right ventricle.

We further explored the protective effects of XYT on CoCl_2_-treated H9c2 cells, which were widely used as cardiomyocyte models of hypoxic injury and apoptosis in vitro [52]. The results showed that XYT could reverse the CoCl_2_-induced decrease of cell viability, while XYT pretreatment alone did not induce any significant cell viability loss. In addition, XYT could reduce the expression of Bax and caspase-3 proteins while increasing the expression of Bcl-2 protein in a concentration-dependent manner after exposure to CoCl_2_-induced hypoxia, which was consistent with the results in vivo. Meanwhile, XYT also lowered the percentage of apoptotic cells. These results indicated that XYT could protect cardiomyocytes from CoCl_2_-induced hypoxic injury and the mechanism is related to its anti-apoptotic effects.

## Conclusions

Our study showed that XYT attenuated chronic hypoxia-induced RV remodeling and fibrosis in RV failure rats. We further demonstrated that XYT was able to protect cardiomyocytes against hypoxia-induced apoptosis in vivo and *vitro* models of hypoxia-induced cardiac injury. The findings propose the promising therapeutic value of XYT in the clinical management of hypoxia-induced cardiac injury.

## Data Availability

All data used/analyzed during the current study are available from the corresponding author on reasonable request.

## Abbreviations

BW: Body weight
Cap: Capopril
CoCl_2_: Cobalt chloride
+ dP/dtmax: The maximal rate of increase of ventricular pressure
-dP/dtmin: The maximal rate of increase decrease of ventricular pressure
ECG: Electrocardiogram
HPH: Hypoxia-induced pulmonary hypertension
HPLC–MS: High-performance liquid chromatography-tandem mass spectrometry
IL-1β: Interleukin-1β
IL-6: Interleukin-6
LV + S: Left ventricle plus interventricular spetum
P Amp: Amplitude of the P wave
QTc interval: Rate-corrected QT interval
RV: Right ventricular
RVEDP: Right ventricular end-diastolic pressure
RVH: Right ventricular hypertrophy
RVHI: Right ventricular hypertrophy index
RVP: Right ventricular pressure
RVSP: Right ventricular systolic pressure
RVW: Right ventricular weight
SD: Sprague–Dawley
SEM: Standard error of the mean
TCM: Traditional Chinese medicine
TNF-α: Tumor necrosis factor-alpha
WA%: The percentage of medial wall area
WT%: The percentage of medial wall thickness
XYT: Xinyang Tablet
